# Supplementary material for: Analysis of PPARγ Signaling Activity in Psoriasis
Source: Int J Mol Sci. 2021 Aug 10;22(16):8603. doi: 10.3390/ijms22168603 (PMC8395241; doi:10.3390/ijms22168603)
Supplement: Supplementary file 1 [file ijms-22-08603-s001.zip › Supplemental materials_Analysis of PPARg signaling activity in psoriasis/Pathway models/Models images and html files/Model of PPARG related pathways in psoriasis (short version)/15449.html]

NOD1


# Protein NOD1

|  |  |
| --- | --- |
| URN | urn:agi-llid:10392 |
| Total Entities | 0 |
| Connectivity | 1213 |
| Name | NOD1 |
| Description | nucleotide binding oligomerization domain containing 1 |
| Notes | This gene encodes a member of the nucleotide-binding oligomerization domain (NOD)-like receptor (NLR) family of proteins. The encoded protein plays a role in innate immunity by acting as a pattern-recognition receptor (PRR) that binds bacterial peptidoglycans and initiates inflammation. This protein has also been implicated in the immune response to viral and parasitic infection. Major structural features of this protein include an N-terminal caspase recruitment domain (CARD), a centrally located nucleotide-binding domain (NBD), and 10 tandem leucine-rich repeats (LRRs) in its C terminus. The CARD is involved in apoptotic signaling, LRRs participate in protein-protein interactions, and mutations in the NBD may affect the process of oligomerization and subsequent function of the LRR domain. Mutations in this gene are associated with asthma, inflammatory bowel disease, Behcet disease and sarcoidosis in human patients. [provided by RefSeq, Aug 2017] |

---

|  |  |
| --- | --- |
| Pathway | NOD-like Receptors |
|  | Proteins Involved in Acne Vulgaris |
|  | Genes with Mutations Associated with Atopic Dermatitis |
|  | Proteins Involved in Helicobacter Infections |
|  | CagA Phosphorylation Independent Signaling |
|  | Dendritic Cell Function in Ulcerative Colitis |
|  | Proteins Involved in Periodontitis |
|  | Blau Syndrome |
|  | Model of PPARG signaling in psoriasis |
|  | PPARG negative regulators and targets |
|  | Model of PPARG related pathways in psoriasis (short version) |
|  | Overview. Diseases of the skin (chapter 11) |
|  | 1\_Insufficient activation of immune response in the middle ear epithelium cells in otitis media |
|  | 1\_Th2 cell response in asthma |
|  | 2\_Paneth cell dysfunction in Crohn's disease |
|  | 1\_Defects in response to pathogens in the gut promotes inflammation in Crohn's disease |
|  | 2\_Intestine epithelial cells drive immunological responses in ulcerative colitis |
|  | 4\_1\_Polymorphisms associated with inflammatory bowel diseases |

---

|  |  |
| --- | --- |
| Group | Genes with Mutations Associated with Atopic Dermatitis |

---

|  |  |
| --- | --- |
| MedScan ID | 10392 |

---

|  |  |
| --- | --- |
| LocusLink ID | 10392 |
|  | 107607 |
|  | 500133 |

---

|  |  |
| --- | --- |
| Alias | nucleotide oligomerization domain protein 1 |
|  | caspase recruitment domain-containing protein 4 |
|  | Nod1 |
|  | nucleotide-binding oligomerization domain containing 1 |
|  | NOD1 signaling adaptor protein |
|  | F830007N14Rik |
|  | nucleotide binding oligomerization domain protein 1 |
|  | F830007N14 |
|  | nucleotide-binding oligomerization domain, leucine rich repeat and CARD domain containing 1 |
|  | NLRC1 |
|  | Nod I |
|  | RGD1562269 |
|  | NLR family, CARD domain containing 1 |
|  | caspase recruitment domain 4 |
|  | RIKEN cDNA F830007N14 gene |
|  | Card4 |
|  | caspase recruitment domain family, member IV |
|  | C230079P11 |
|  | CLR7.1 |
|  | caspase recruitment domain family, member 4 |
|  | nucleotide-binding oligomerization domain-containing protein 1 |

---

|  |  |
| --- | --- |
| GO ID | 0005524 |
|  | 0050700 |
|  | 0008656 |
|  | 0042834 |
|  | 0042803 |
|  | 0044877 |
|  | 0007254 |
|  | 0000187 |
|  | 0006919 |
|  | 0006915 |
|  | 0071225 |
|  | 0006952 |
|  | 0050830 |
|  | 0042742 |
|  | 0016045 |
|  | 0009595 |
|  | 0006954 |
|  | 0045087 |
|  | 0070498 |
|  | 0042228 |
|  | 0035556 |
|  | 0070423 |
|  | 0070374 |
|  | 0043123 |
|  | 0046330 |
|  | 0051092 |
|  | 1901224 |
|  | 0043280 |
|  | 0002606 |
|  | 0032731 |
|  | 0032755 |
|  | 0051000 |
|  | 0032760 |
|  | 1904417 |
|  | 0051259 |
|  | 0007165 |
|  | 0016324 |
|  | 0016323 |
|  | 0005737 |
|  | 0005829 |
|  | 0000166 |
|  | 0002376 |
|  | 0010942 |
|  | 0032874 |
|  | 0042981 |
|  | 0016020 |
|  | 0005886 |
|  | 0002755 |
|  | 0002756 |
|  | 0035666 |
|  | 0035872 |
|  | 0051403 |
|  | 0034166 |
|  | 0034134 |
|  | 0034138 |
|  | 0034142 |
|  | 0034146 |
|  | 0034162 |
|  | 0038123 |
|  | 0038124 |
|  | 0002224 |
|  | 0008063 |
|  | 0006917 |
|  | 0034130 |
|  | 0005622 |

---

|  |  |
| --- | --- |
| KEGG ID | hsa:10392 |
|  | mmu:107607 |
|  | rno:500133 |

---

|  |  |
| --- | --- |
| Organism | Homo sapiens {Organism urn:agi-taxid:9606} |
|  | Mus musculus {Organism urn:agi-taxid:10090} |
|  | Rattus norvegicus {Organism urn:agi-taxid:10116} |
|  | Homo sapiens |
|  | Mus musculus |
|  | Rattus norvegicus |

---

|  |  |
| --- | --- |
| Mouse chromosome position | 6 |

---

|  |  |
| --- | --- |
| OMIM ID | 605980 |

---

|  |  |
| --- | --- |
| Rat chromosome position | 4q24 |

---

|  |  |
| --- | --- |
| Hugo ID | 16390 |
|  | HGNC:16390 |

---

|  |  |
| --- | --- |
| Human chromosome position | 7p14.3 |
|  | 7p15-p14 |

---

|  |  |
| --- | --- |
| Swiss-Prot Accession | A0A024RA73 |
|  | Q9Y239 |
|  | G3XAL1 |
|  | Q9Y239.1 |
|  | Q540J8 |
|  | Q8BHB0 |
|  | Q8BHB0.1 |
|  | D4ADT7 |
|  | B4DTU3 |
|  | Q549U4 |
|  | Q8IWF5 |
|  | Q8BUT6 |

---

|  |  |
| --- | --- |
| GenBank ID | NC\_000007 |
|  | NR\_149002 |
|  | XR\_002956406 |
|  | XR\_926908 |
|  | XR\_001744529 |
|  | XR\_926909 |
|  | XR\_926910 |
|  | XR\_001744530 |
|  | NM\_001354849 |
|  | NP\_001341778 |
|  | XM\_011515081 |
|  | XP\_011513383 |
|  | XM\_005249572 |
|  | XP\_005249629 |
|  | NM\_006092 |
|  | NP\_006083 |
|  | XM\_011515079 |
|  | XP\_011513381 |
|  | XM\_011515083 |
|  | XP\_011513385 |
|  | XM\_011515085 |
|  | XP\_011513387 |
|  | XM\_011515084 |
|  | XP\_011513386 |
|  | XM\_005249568 |
|  | XP\_005249625 |
|  | XM\_006715633 |
|  | XP\_006715696 |
|  | XM\_011515080 |
|  | XP\_011513382 |
|  | XM\_005249576 |
|  | XP\_005249633 |
|  | XM\_017011674 |
|  | XP\_016867163 |
|  | XM\_011515087 |
|  | XP\_011513389 |
|  | XM\_011515088 |
|  | XP\_011513390 |
|  | NG\_013025 |
|  | AACC02000087 |
|  | EAL24453 |
|  | AC005154 |
|  | AC006027 |
|  | AAS46897 |
|  | AH007965 |
|  | AX082205 |
|  | CAC33156 |
|  | CH471073 |
|  | EAW93944 |
|  | EAW93945 |
|  | EAW93946 |
|  | EAW93947 |
|  | EAW93948 |
|  | EAW93949 |
|  | AA845215 |
|  | AF113925 |
|  | AAD28350 |
|  | AF126484 |
|  | AAD29125 |
|  | AK023969 |
|  | BAG51242 |
|  | AK300367 |
|  | BAG62105 |
|  | AY128682 |
|  | AAM98064 |
|  | AY128683 |
|  | AAM98065 |
|  | AY128684 |
|  | AAM98066 |
|  | AY128685 |
|  | AAM98067 |
|  | AY128686 |
|  | AAM98068 |
|  | AY187249 |
|  | AY187250 |
|  | BC020114 |
|  | BC040339 |
|  | AAH40339 |
|  | CV569884 |
|  | KU178514 |
|  | ALQ33972 |
|  | KU178515 |
|  | ALQ33973 |
|  | KU178516 |
|  | ALQ33974 |
|  | KU178517 |
|  | ALQ33975 |
|  | Q9Y239 |
|  | NC\_000072 |
|  | NM\_172729 |
|  | NP\_766317 |
|  | NM\_001171007 |
|  | NP\_001164478 |
|  | XM\_006505278 |
|  | XP\_006505341 |
|  | XR\_377400 |
|  | AC079183 |
|  | CH466597 |
|  | EDK98713 |
|  | AI645572 |
|  | AK082663 |
|  | BAC38566 |
|  | AK089662 |
|  | BAC40940 |
|  | AK137585 |
|  | BAE23420 |
|  | AY160222 |
|  | AAN52479 |
|  | BC043670 |
|  | AAH43670 |
|  | Q8BHB0 |
|  | NC\_005103 |
|  | NM\_001109236 |
|  | NP\_001102706 |
|  | XM\_008762910 |
|  | XP\_008761132 |
|  | AC\_000072 |
|  | AABR07060610 |
|  | AAHX01030269 |
|  | AAHX01030270 |
|  | CH474011 |
|  | EDL88106 |
|  | XM\_011515086 |
|  | XP\_011513388 |
|  | XM\_011515082 |
|  | XP\_011513384 |
|  | XR\_926907 |
|  | NC\_018918 |
|  | AF149773 |
|  | AF149774 |
|  | AAD43922 |
|  | AMYH02015456 |
|  | AMYH02015457 |
|  | AMYH02015458 |
|  | AMYH02015459 |
|  | AMYH02015460 |
|  | XM\_006505277 |
|  | XP\_006505340 |
|  | AC\_000028 |
|  | NW\_001073834 |
|  | AAHY01053426 |
|  | AAHY01053427 |
|  | AAHY01053428 |
|  | AAHY01146467 |
|  | BC042670 |
|  | AAH42670 |
|  | XM\_006715635 |
|  | XP\_006715698 |
|  | XM\_005249575 |
|  | XP\_005249632 |
|  | XM\_005249574 |
|  | XP\_005249631 |
|  | XM\_005249573 |
|  | XP\_005249630 |
|  | XM\_006715634 |
|  | XP\_006715697 |
|  | XR\_428069 |
|  | XM\_006715636 |
|  | XP\_006715699 |
|  | XM\_006715637 |
|  | XP\_006715700 |
|  | AC\_000139 |
|  | ABBA01056813 |
|  | AC\_000068 |

---

|  |  |
| --- | --- |
| Swiss-Prot ID | NOD1\_HUMAN |
|  | NOD1\_MOUSE |

---

|  |  |
| --- | --- |
| Cell Localization | Cytoplasm |
|  | Cell membrane |
|  | Apical cell membrane |
|  | Basolateral cell membrane |

---

|  |  |
| --- | --- |
| Ensembl ID | ENSG00000106100 |
|  | ENSP00000222823.4 |
|  | ENST00000222823.9 |
|  | ENSP00000416946.1 |
|  | ENST00000434755.5 |
|  | ENSMUSG00000038058 |
|  | ENSMUSP00000130487.1 |
|  | ENSMUST00000168172.3 |
|  | ENSMUSP00000055747.8 |
|  | ENSMUST00000060655.14 |
|  | ENSRNOG00000010629 |
|  | ENSRNOP00000014324.4 |
|  | ENSRNOT00000014324.6 |
|  | ENSP00000222823 |
|  | ENST00000222823 |
|  | ENSP00000416946 |
|  | ENST00000434755 |
|  | ENSMUSP00000130487 |
|  | ENSMUST00000168172 |
|  | ENSMUSP00000055747 |
|  | ENSMUST00000060655 |
|  | ENSRNOP00000014324 |
|  | ENSRNOT00000014324 |

---

|  |  |
| --- | --- |
| MGI ID | MGI:1341839 |
|  | 1341839 |

---

|  |  |
| --- | --- |
| RGD ID | 1562269 |

---

|  |  |
| --- | --- |
| Unigene ID | Hs.405153 |
|  | Hs.738731 |
|  | Mm.28498 |
|  | Rn.53929 |

---

|  |  |
| --- | --- |
| Homologene ID | 4440 |

---

|  |  |
| --- | --- |
| IPI ID | IPI00005776 |
|  | IPI00221820 |

---
